# Self-management versus usual care for greater trochanteric pain syndrome (the HIPS trial): study protocol for a randomised controlled trial

**DOI:** 10.1136/bmjopen-2024-090688

**Published:** 2025-04-05

**Authors:** Thea Morin Melås, John Bjørneboe, Niels Gunnar Juel, Maren Lunder Wefring, Sigrid Skatteboe, Rikke Munk Killingmo, Kaia B Engebretsen, Michael Skovdal Rathleff, Britt Elin Øiestad, Helene Lundgaard Søberg, Are Hugo Pripp, Jens Ivar Brox, Marianne Bakke Johnsen

**Affiliations:** 1 Department of Rehabilitation Science and Health Technology, Oslo Metropolitan University, Oslo, Norway; 2 Department of Physical Medicine and Rehabilitation, Oslo University Hospital, Oslo, Norway; 3 Department of Health Science and Technology, Faculty of Medicine, Aalborg University, Aalborg, Denmark; 4 Department of Physical Therapy and Occupational Therapy, Aalborg University Hospital, Aalborg, Denmark; 5 Oslo University Hospital, Oslo, Norway

**Keywords:** PAIN MANAGEMENT, Patient-Centered Care, REHABILITATION MEDICINE, Clinical Trial, Self-Management

## Abstract

**Introduction:**

Greater trochanteric pain syndrome (GTPS) is a common and disabling condition characterised by lateral hip pain. The condition often persists for several months, and there is low evidence for any superior treatment. The aim of this study protocol is to describe a randomised controlled trial (RCT) investigating the effectiveness of a self-management programme versus usual care for patients with GTPS.

**Methods and analysis:**

The study is designed as an observer-blinded, parallel group, superiority RCTcomparing a self-management programme (n=55) with usual care (n=55). Eligible patients with GTPS will be included based on reproduction of pain on palpation in the greater trochanteric region and at least one positive clinical provocation test. The self-management programme includes 3–5 individual sessions with a physiotherapist over 12 weeks, addressing physical, emotional and behavioural factors deemed relevant by the patient. Usual care will receive general information about GTPS, activity management and are free to seek further treatment in primary care as wanted. The primary outcome measure is the Norwegian version of the Victorian Institute of Sports Assessment for gluteal tendinopathy questionnaire (VISA-G-Norwegian). Outcomes will be assessed at baseline, 3, 6 and 12 months. A longitudinal mixed effects model will be used to assess the effectiveness of treatment on pain and disability across all time points, with the primary endpoint at 6 months. Cost-effectiveness will be expressed by mean incremental cost-effectiveness ratios (ICERs) from a societal and healthcare perspective. Bootstrapping will be used to estimate ICER uncertainty.

**Ethics and dissemination:**

The Norwegian Regional Committees for Medical and Health Research Ethics have approved the project (2023/590816), and it will be in accordance with recommendations from the Data Inspectorate at Oslo University Hospital (22/26396). The results from the study will be disseminated through publications in peer-reviewed journals, in conference presentations and through the user representative.

**Trial registration number:**

NCT06297148.

**STRENGTHS AND LIMITATIONS OF THIS STUDY**

- Preplanned evaluation of implementation fidelity for the self-management intervention.
- The first randomised controlled trial investigating self-management for greater trochanteric pain syndrome.
- Collection of data at multiple time points enables evaluation over time.
- Blinding of therapists and participants is not possible.
- Only self-reported outcome measures.

## Introduction

 Greater trochanteric pain syndrome (GTPS) is a persistent and disabling condition, characterised by pain on the lateral side of the hip.^1 2^ It serves as an umbrella term for soft-tissue pathology, including trochanteric bursitis and degenerative tendinopathies.^3^,^5^ The prevalence and incidence rates have been reported to be 4.2 and 3.3, respectively, per 1000 person-years in the general practice population,^1^ and the condition is most common in women during their fourth to sixth decades of life.^3^ People with GTPS report disturbed sleep, limitations in daily function and reductions in work participation.^2 6^ Greater severity of pain and disability is associated with lower levels of physical activity and greater psychological distress, including higher pain catastrophising, depression and lower self-efficacy.^7^ The quality of life has been compared with patients with severe hip osteoarthritis (OA) awaiting hip surgery.^6^ The prognosis is considered to be poor, and one in every three will continue to experience symptoms 1–5 years after the initial diagnosis.^8^

A range of non-operative treatment options have been investigated for GTPS.^9^,^11^ Most trials investigated passive modalities such as injection and shockwave therapy. However, a recent systematic review recommends an active approach with exercise as the first-line treatment.^11^ Over the last decade, there has been a shift towards exercise and education as the cornerstone of management.^12 13^ This combination has been shown to be superior to both injection therapy and wait-and-see.^14^ However, uncertainty exists about which content and delivery modes provide the best outcomes, and non-specific (sham) exercises have been shown to be equally effective to specific hip exercises in combination with patient education.^15^ This question if general activity is sufficient, with education suggested to be an even more important component to be explored further in future research.^15^ Patients with GTPS report low self-efficacy and uncertainty about how to interpret pain related to activity,^16 17^ making it challenging to find the right balance between physical activity and staying active. Given the persistent nature and impact of GTPS, any form of treatment should include both physical and psychosocial aspects and incorporate self-management strategies. Such strategies can support the patient to better manage ongoing challenges related to their condition, which may prove beneficial in improving both pain and disability. Similar interventions have been feasible and acceptable for other types of musculoskeletal disorders,^18^,^21^ but have yet to be investigated for patients with GTPS. The main objective of this study protocol is to describe a randomised controlled trial (RCT) comparing the effectiveness of a self-management programme versus usual care for patients with GTPS. The primary hypothesis is that pain and disability outcomes will be superior in the self-management group compared with usual care after 6 months. Secondary objectives will be to evaluate implementation fidelity, explore potential predictors, and conduct mediation and cost-effectiveness analyses.

## Methods and analysis

The study will have a parallel group, superiority RCT design, comparing self-management versus usual care.

### Participants

A total of 110 participants aged 18–70, with a history of lateral hip pain >3 months, and a verified clinical GTPS diagnosis will be included. A cut-off in pain intensity of at least 3 on a numeric rating scale (NRS) was set to ensure the recruitment of patients who are currently experiencing symptoms. All participants need to understand oral and written Norwegian, to be able to answer self-reported questionnaires. Further details on inclusion and exclusion criteria are outlined in box 1.

### Recruitment

**Figure 1:**
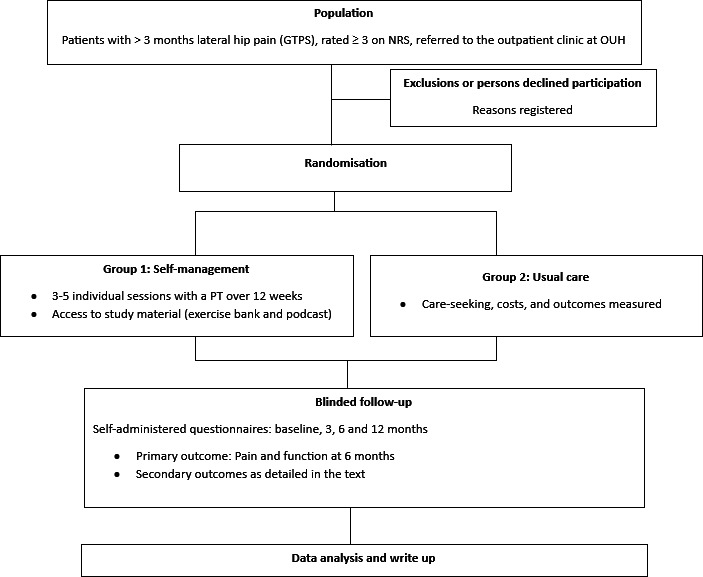
Flowchart of the trial. GTPS, greater trochanteric pain syndrome; OUH, Oslo University Hospital.

Participants will be recruited from the outpatient clinic at the Department of Physical Medicine and Rehabilitation at Oslo University Hospital (OUH), Norway. Annually, approximately 120 patients in our department are diagnosed with GTPS. Patients are referred to the clinic by general practitioners, other hospital departments or external therapists such as manual therapists or chiropractors. All relevant collaborators will be informed about recruitment to the study both prior to and during inclusion. After referral, a medical doctor at the outpatient clinic will conduct a standard clinical consultation. If GTPS is diagnosed, the patient will be informed about the study and asked if they are willing to participate. A medical doctor (MLW) dedicated to the study will then screen patients for eligibility by conducting the baseline assessment. If eligible for inclusion, more detailed information will be given by a physiotherapist (TMM). A flowchart of the study is outlined in figure 1. Recruitment to the study started on 12 March 2024. The primary completion date for recruitment is estimated to be 1 July 2026, and the final study completion date for the 12-month follow-up is estimated to be 30 June 2027.

### Baseline assessment

A clinical assessment will be conducted prior to group allocation with the objective of excluding any differential diagnosis outlined in the exclusion criteria (see box 1) and confirming the diagnosis of GTPS. This includes pain on palpation of the greater trochanter area in conjunction with a positive result (reproduction of the participant’s known lateral hip pain) on at least one of the following clinical provocation tests: Single Leg Stance test*,* Hip Flexion, Adduction, External rotation test (FADER), FADER with Isometric Internal rotation Resistance (FADER-R), Passive Hip Adduction test (ADD), ADD with Resisted Isometric Abduction (ADD-R) and Hip Flexion, Abduction, External Rotation test (FABER). This combination of clinical provocation tests has been used in a prior RCT study for the current patient population.^22^ The clinical provocation tests are described in detail in online supplemental file 1. In addition, muscle strength during abduction will be measured according to an established protocol, using a Mustec HD handheld dynamometer on both sides. In instances where MRI has been performed, findings will be registered for descriptive purposes, but MRI scans are not a prerequisite for inclusion in the study. A supplementary radiological examination will be performed to exclude other pathology, if relevant, based on the clinical examination.

Box 1 **Inclusion and exclusion criteria**

**Inclusion criteria**

- Age between 18 and 70 years.
- History of lateral hip pain >3 months.
- Reported average pain intensity last week ≥3 on a numeric rating scale (NRS).
- Lateral hip pain is the main complaint.
- Pain on palpation of the greater trochanter region.
- Reproduction of lateral hip pain on at least one clinical provocation test (*SLS, FADER, FADER-R, ADD, ADD-R *and *FABER*).

**Exclusion criteria**

- Significant back pain causing referred pain to the lateral hip.
- Clinical signs of radiculopathy.
- Symptomatic hip osteoarthritis (OA).
- Other hip joint pathologies.
- History of surgery or trauma on the affected side.
- Pregnancy or postpartum pelvic pain (under 12 months since birth).
- Active cancer.
- Not able to write, read and comprehend Norwegian (without the use of an interpreter).

SLS, Single Leg Stance; FADER, Flexion, Adduction, External Rotation test; FADER-R, FADER with Isometric Resistance against Internal Rotation; ADD, Adduction test; ADD-R, ADD with Isometric Resistance to Abduction; FABER, Flexion, Abduction, External Rotation testFlexion,adduction,externalrotation test..

### Randomisation

Participants will be randomly allocated into one of the two groups: (1) self-management programme or (2) usual care. They will receive oral and written information prior to enrolment that they have an equal chance of being allocated into either group. They are informed that the two treatments given are potentially equally effective. The randomisation is concealed, and the sequence is computer-generated with blocks of variable size. The randomisation list is stored in a separate file on a research server, only available to a separate researcher not involved in the delivery of the interventions or the outcome assessments. Information about group allocation will be given by telephone from a physiotherapist dedicated to the study (TMM). The physiotherapists delivering the self-management intervention will not be involved in, or have contact with, the primary healthcare service treating the usual care group (the comparator), to avoid cross-contamination between the two groups.

### Interventions

Participants in both groups will receive standard information about the condition and advice concerning physical activity, as is the usual practice at the hospital department. A pamphlet with a summary of this information is handed out to all participants at baseline, prior to randomisation. Participants who are allocated to the intervention group will be treated by physiotherapists at OUH. The comparator group is free to choose if they want further follow-up and will do so in primary care. The risk of adverse effects is low in both the self-management programme and usual care, which include treatments commonly used for musculoskeletal complaints. However, should any adverse events occur, the participants or treating physiotherapists will report to the principal investigator who will record the event and ensure that proper treatment for the adverse event is undertaken.

#### Group 1: self-management

The self-management programme in the current study is based on the frequently used theoretical framework described by Lorig and Holman.^23^,^25^ The programme aims to equip the patients with the necessary skills to actively participate and take responsibility in the management of their GTPS. The intervention targets to improve function, reduce avoidance behaviours (if relevant) and build confidence to re-engage in activities that are meaningful to the patient. How the patient perceives the problem is essential,^26^ and confidence in their ability to handle specific tasks or challenges (self-efficacy) is considered a key component,^27^ as enhancement of self-efficacy may contribute to facilitate self-management. The self-management intervention has been developed through a structured approach and in collaboration with the user representative. The description of the programme in the current protocol is in accordance with the Criteria for Reporting the Development and Evaluation of Complex Interventions checklist (CReDECI 2). The last stages of this checklist will be completed when reporting the results of the study.

The intervention will consist of 3–5 individual sessions over 12 weeks, each session lasting up to 60 min. The timing of and the number of sessions will be at the discretion of the physiotherapist in discussion with the participant. The sessions will include individually tailored education and address physical, cognitive and behavioural factors deemed as relevant according to concerns, challenges and prior experiences reported by the participant. The focus will be on identifying and utilising resources, through the five core skills of self-management; problem-solving, decision-making, resource utilisation, therapeutic alliance and taking action.^23^ The operationalisation of the core skills and main themes is outlined in table 1. Session one will include problem-solving related to difficulties in activity, exercise, daily life or work life, forming a therapeutic alliance. Important aspects will be to establish a common understanding of the diagnosis and identify individual challenges and resources. Goals that are specific, measurable, achievable, relevant and time-bound (SMART) will be established. An activity plan between sessions will be used as an exposure to maintain, change or create health behaviour. Session two will focus on the decision-making process, eg, how to implement behaviour and deal with pain or fears. Experiences with the activity plan will be discussed, and barriers and facilitators identified and explored. Current coping strategies, concerning both emotional and behavioural responses, will be addressed in addition to physical activity behaviour. Relevant feedback and helpful information are given by the physiotherapist. The activity plan is revised and adjusted accordingly. Session three will include resource utilisation, teaching the participants how to use available resources to adhere to the behavioural change going forward. Reconceptualising unhelpful thoughts, pain beliefs and behaviours may also be relevant to address during this session. Sessions four and five will be offered as additional consultations, within the 12-week timeframe. Relevant topics will be revisited, depending on the needs of the participant. Participants in the self-management group will have access to exercise programmes and a podcast. The podcast consists of three episodes where information from the treatment sessions is reinforced, for example, about the GTPS diagnosis, self-management and pain management strategies. Participants are allowed to continue with their usual medication but will be asked not to receive any other treatment for GTPS during the 12-week intervention period.

**Table 1:** Main themes and operationalisation of the self-management skills

Themes	Content and material	Self-management skills
Session 1		
Diagnostic understanding	Patient beliefs	Therapeutic allianceProblem-solvingDecision-makingTaking action
Individual challenges and resources	SMART goalsActivity plan	
Session 2		
Coping strategies (*behavioural and emotional response*)	Reflection from activity planReorientation of goal/plan	Therapeutic allianceProblem-solvingDecision-makingResource utilisationTaking action
Physical activity behaviour		
Session 3		
Beliefs about pain	Reflection from activity planReorientation of goal/plan	Therapeutic allianceProblem-solvingDecision-makingResource utilisationTaking action
Health behaviour		

SMARTspecific, measurable, achievable, relevant and time-bound

#### Group 2: usual care

The comparator will be usual care as directed by the normal assessment and clinical decision-making by the treating medical doctor in the outpatient clinic. Usual care consists of diagnostics and information about the condition given during the first consultation at OUH, in addition to general advice on pain management and physical activity given at the baseline assessment. Any future follow-up in primary care will be decided by the participant and their healthcare providers, for example, general practitioner, physiotherapist, chiropractor or other healthcare professionals. Details on the nature of the treatment (type, frequency, etc) will be collected through the self-administered digital questionnaires at 3, 6 and 12 months.^28^ All participants will be asked to report any contact with the healthcare service, including what type of health professional they have visited, the number of sessions and the treatment that was given. The number of sick days related to GTPS and any changes regarding sick leave will also be accounted for through self-report (Institute for Medical Technology Assessment, iMTA, productivity cost questionnaire).^29^

### Training of physiotherapists

Three physiotherapists from OUH, all experienced within the field of longstanding musculoskeletal pain, will deliver the self-management intervention. The physiotherapists have all been actively involved in the development of the programme, including discussions on the operationalisation of the five core skills of self-management and the identification of relevant themes to address. They attended regular meetings with the project manager from 6 months prior to the study (in total 7 hours), where they discussed communication tools, how to address relevant themes and the use of study material regarding GTPS, pain management, coping strategies and behavioural change. To ensure that the intervention was delivered per protocol, a semistructured treatment guide has been developed. This guide contains standardised illustrations to use during consultations, possible prompts and relevant resource literature. In addition, the physiotherapists each had 1–5 test patients prior to inclusion. Experiences and challenges from these sessions were discussed and used to adjust the intervention content.

### Treatment fidelity assessment

Implementation fidelity of the self-management intervention will be evaluated based on the conceptual framework by Carroll *et al*,^30^ including adherence and potential moderators. Adherence will be measured by evaluating (1) content (active ingredients of the intervention), (2) coverage (number of potential participants included), (3) frequency and (4) duration of sessions according to the established protocol. Potential moderators are aspects that may influence the adherence to the intervention as well as the outcome. These include participant responsiveness, comprehensiveness of policy descriptions, strategies to facilitate implementation, quality of delivery, recruitment and the context in which the intervention is delivered.^30^ Sources of data collection will be self-reported checklists, simple logs from meetings and observations of consultations. Monthly meetings with the physiotherapists and researchers will be scheduled to discuss any challenges during the study period. The intention of regular discussions is to ensure that the physiotherapists are delivering as close to the same intervention as possible. However, as the intervention is patient-centred and individualised, a pragmatic approach with flexibility within certain established boundaries is allowed. Simple logs from the meetings will be used to identify common barriers and facilitators in delivering the intervention. After each consultation, the physiotherapists will fill out a self-reported checklist to evaluate if treatment has been delivered according to protocol. The checklist includes practical elements of the session, which themes have been addressed, the establishment of SMART goals and an activity plan, and if the patient has completed the activity plan as agreed. In addition, an external observer will attend one consultation in 10% of the participants. The observer will fill out the same checklist as the physiotherapist, giving the opportunity to compare the level of agreement according to the protocol. The number of observations will be distributed equally among the physiotherapists delivering the intervention and conducted during sessions one and two. After the final consultation, participants will be asked to fill out a self-report checklist concerning their perception of the content of the intervention, to what extent they felt involved in the process of setting goals and making an activity plan, utilisation of the study material and to what degree they have implemented any of the elements they have learnt.

### Data collection and outcome measures

All data collection will be self-reported and electronically administered through web-based questionnaires^28^ with a secure digital identification protocol that communicates directly with services for sensitive data where the data is stored.^31^ Before the study started, the web-based questionnaire package was tested on 11 patients at the outpatient clinic. They gave feedback on time consumption, layout and comprehensibility. Data in the study will be collected at baseline, 3, 6 and 12 months. A printed version will be available for participants who have difficulty answering the web-based questionnaires. Participant demographics such as age, sex, education, work status, comorbidities, physical activity level, sleep disturbance and menopausal status are included at baseline to describe the patient population. Link to questionnaires at 3, 6 and 12 months will be sent by email and SMS. If participants do not complete the questionnaires within a week, a reminder will be sent by SMS. A final reminder will be given by telephone if the participant has not answered within 3 days of the latest reminder.

#### Primary outcome

The primary outcome will be the Victorian Institute of Sports Assessment for gluteal tendinopathy questionnaire (VISA-G-Norwegian).^32^ This is a diagnosis-specific and patient-reported questionnaire, consisting of eight questions assessing the severity of pain and disability. Total score ranges from 0 to 100, with higher scores indicating less pain and disability. The questionnaire is validated for the current patient population and is frequently used in research, giving the opportunity to compare results.^33^ VISA-G-Norwegian has demonstrated good test–retest reliability, moderate internal consistency, and no floor or ceiling effects.^32^

#### Secondary outcomes

Secondary outcomes are depicted in table 2 and include pain intensity and distribution, function, health-related quality of life, psychological distress, pain-related self-efficacy, patients expected change in pain and function, healthcare utilisation, productivity loss, perceived change in lateral hip pain from baseline to follow-up at 6 months and patient accepted symptom state.

**Table 2:** Timeline of measurements

Measure	Purpose	Baseline	3 months	6 months	12 months
Primary outcome					
VISA-G-Norwegian^32^	Evaluate the effectiveness of the intervention	X	X	X	X
Secondary outcomes					
NRS^40^	Pain intensity and function	X	X	X	X
Number of painful sites^41^	Pain distribution	X	X	X	X
EQ-5D-5L^42^	Evaluate health-related quality of life	X	X	X	X
HSCL-10^43^	Psychological distress (potential mediator)	X	X	X	X
PSEQ^44^	Pain-related self-efficacy (potential mediator)	X	X	X	X
Expectations NRS	Expected change in NRS pain and function	X	X	X	
Expectations VISA-G-Norwegian	Expected change in VISA-G score from baseline to 6 months	X			
iMTA iMCQ^45^	Cost-effectiveness of the intervention	X	X	X	X
iMTA iPCQ^29^	Cost-effectiveness of the intervention	X	X	X	X
Global Rating of Change scale^46^	Patient perceived a change in lateral hip pain			X	
Patient accepted symptom state^47^	Patient satisfaction with current symptom state			X	

EQ-5D-5LEuroQoL-5 dimensions-5 levelHSCL-10Hopkins Symptoms Checklist-10iMCQMedical Consumption QuestionnaireiPCQProductivity Cost QuestionnaireNRSnumeric rating scalePSEQPain Self-Efficacy QuestionnaireVISA-G-NorwegianVictorian Institute of Sports Assessment for gluteal tendinopathy questionnaire

### Sample size

Based on previous RCTs^14 15 34^ and validation of the primary outcome, the VISA-G-Norwegian,^32^ sample size in the current RCT was calculated using an SD of 17 and a mean difference of 10 points between groups. This resulted in a sample size of 94 participants (ie, 47 in each group), assuming 80% power and Alpha of 0.05. To account for dropout (15%), we will recruit 110 participants (55 per group).

### Blinding

Due to the nature of therapeutic studies, blinding of the physiotherapists and participants will not be possible. However, the statistician and researchers handling the data will be blinded regarding treatment allocation. Thus, this will be an observer-blinded RCT. Participants, and physiotherapists delivering the intervention, will not be informed about any anticipated results.

### Statistical analysis

Statistical analysis will be conducted blinded using the intention-to-treat principle. Superiority hypothesis testing will be performed to test the effectiveness of the self-management programme compared with usual care. The superiority of self-management over usual care will be claimed if the two-sided p value is less than 5% in the test comparing the mean difference between groups at 6 months in VISA-G-Norwegian score. A statistical analysis plan (SAP) was developed in collaboration with a statistician (AHP) dedicated to the study.^35^

#### Treatment effectiveness analysis

The primary analysis will compare the two intervention groups (self-management versus usual care) on their mean difference in pain and disability (VISA-G-Norwegian score) at baseline, 3 and 6 months. The estimated mean difference between groups at 6 months (main endpoint) will be analysed using a longitudinal mixed effects model analysis of covariance (ANCOVA).^36^ The model will include the fixed effects of time, intervention, the interaction between time and intervention, and the outcome variable at baseline as a covariate with a subject-specific random intercept. Secondary outcomes assessed at multiple time points (baseline, 3, 6 and 12 months) will be analysed by the same approach as described for the primary outcome, using the intention-to-treat and the per-protocol approaches. The baseline score will be included as a covariate in all the analyses.

#### Mediation analysis

Causal mediation analysis^37^ will be conducted to explore the causal pathway between treatment allocation and the primary outcome of pain and disability. Pain self-efficacy and emotional distress will be considered as potential mediators that may be part of the causal pathway between intervention and outcome.^7^ A separate SAP will be published (https://clinicaltrials.gov/), including graphic representation (eg, directed acyclic graphs) to depict the assumed associations between treatment, mediators, outcome and possible confounders.^38^

#### Prediction analysis

We will perform exploratory analysis to evaluate factors that may predict differential treatment effects for a subgroup of participants at 12 months follow-up. Multivariable logistic and linear regression analysis will be used to explore predictive factors for the primary outcome; pain and disability (VISA-G-Norwegian). Model building will be done in a way that is appropriate for the given sample sizes, by restricting the number of potential predictive factors and considering shrinkage methods to stabilise predictions.^39^ A more detailed protocol and SAP for the prognostic study will be outlined.

#### Cost-effectiveness analysis

Costs related to healthcare utilisation per patient will be estimated by multiplying the frequency of use by unit costs collected from national pricelists. Cost related to productivity loss per patient will be estimated by multiplying the number of workdays with productivity loss by an estimated average wage rate including taxes and social costs from Statistics Norway. Differences between groups in cost will be described with means (95% CI) and evaluated with Student’s t-test. Differences between groups in Quality Adjusted Life-Year will be described with means (95% CI) and evaluated with ANCOVA to adjust for baseline scores unequally distributed across the groups at baseline. Cost-effectiveness will be estimated with mean incremental cost-effectiveness ratios (ICERs) by dividing mean differences in costs by mean differences in effects. Bootstrapping will be used to estimate ICER uncertainty.

### Patient and public involvement

User involvement was embedded through an established patient panel at OUH from the beginning of this project. The user representative gave the following input to the protocol: contributed with feedback on the content of the self-management intervention and relevant themes to address, participated in the creation of study material including the pamphlet, podcast and standardised illustrations to use during the self-management sessions, contributed to discussions about which questionnaires to include and gave feedback on the burden of data collection in the study. The user representative will play a key role in the dissemination of project results to relevant stakeholder communities, including formulation and distribution of patient-friendly summaries.

## Ethics and dissemination

The project will adhere to the principles of the Declaration of Helsinki. Participation requires a signed informed consent (see online supplemental file 2), which will be collected by the medical doctor conducting the baseline assessment (MLW). The study protocol, different interventions and data collection are explained in a consent form. Ethical approval has been obtained from the Regional Committees for Medical and Health Research Ethics (2023/590816). The protocol for the study is in accordance with the Standard Protocol Items: Recommendations for International Trials 2013 guidelines and registered with the ClinicalTrials.gov database (NCT06297148). Any major changes in the study will be reported to the Regional Committees for Medical and Health Research Ethics and documented in the ClinicalTrials register. Authorship will be based on the Vancouver Convention. Results will be disseminated in peer-reviewed scientific journals, in relevant conferences, in popular press articles and on social media to scientific and general public audiences. Results from the proposed project may have valuable short- and long-term effects for the individual and society. It may change the type and mode of treatment and guide the content of care for GTPS patients, relevant to both primary and secondary care.

## supplementary material

10.1136/bmjopen-2024-090688 online supplemental file 1

10.1136/bmjopen-2024-090688 online supplemental file 2
