# Supplementary material for: Self-management versus usual care for greater trochanteric pain syndrome (the HIPS trial): study protocol for a randomised controlled trial
Source: BMJ Open. 2025 Apr 5;15(4):e090688. doi: 10.1136/bmjopen-2024-090688 (PMC11973791; doi:10.1136/bmjopen-2024-090688)
Supplement: online supplemental file 1 [file bmjopen-15-4-s001.pdf]

Supplementary 1

Baseline assessment

Provocation tests for confirming the clinical diagnosis of GTPS:

1. Single Leg Stance test (SLS)

One leg is raised to approximately 60-90° hip flexion. The position is maintained for a maximum of 30 seconds. If necessary, the participant is allowed to stand next to a wall, with the option of utilizing one finger for support against the wall to keep balance. The test is considered positive if the participant reports pain in the trochanteric region within the time limit.

2. Hip Flexion-Adduction-External rotation test (FADER)

In a supine position, the hip of the participant is passively brought into 90° of flexion, adducted and externally rotated to the end range of motion. Positive if well-known lateral hip pain is reproduced.

3. FADER with Isometric Internal rotation Resistance (FADER-R)

While in the end range of the FADER position, the participant is instructed to resist an externally directed rotational force, thereby performing an isometric internal rotation. Positive if well-known lateral hip pain is reproduced.

4. Passive Hip Adduction test (ADD)

The participant lies on their side with the bottom hip and knee flexed at approximately 60° (hip) and 90° (knee). The examiner supports the upper leg, which is extended at the knee and neutrally rotated, aligning the femur with the truncus. The anterior superior iliac spines are vertically aligned in the frontal plane. Then the examiner adducts the leg in the frontal plane to its maximal range while stabilizing the pelvis. Positive if well-known lateral hip pain is reproduced.

5. ADD with Resisted Isometric Abduction (ADD-R)

From the end position of the ADD test, the participant is asked to elevate the thigh against the examiner’s hand, providing resistance. Positive if well-known lateral hip pain is reproduced.

6. Hip Flexion-Abduction-External rotation test (FABER)

With the participant in supine, the lateral malleolus of the examined leg is positioned above the patella on the contralateral leg. The examiner stabilizes the pelvis at the anterior superior iliac spine, while passively lowering the knee, which induces abduction and external rotation of the hip. Positive if well-known lateral hip pain is reproduced.
