# Supplementary material for: Self-management versus usual care for greater trochanteric pain syndrome (the HIPS trial): study protocol for a randomised controlled trial
Source: BMJ Open. 2025 Apr 5;15(4):e090688. doi: 10.1136/bmjopen-2024-090688 (PMC11973791; doi:10.1136/bmjopen-2024-090688)
Supplement: online supplemental file 2 [file bmjopen-15-4-s002.pdf]

Oslo universitetssykehus

versjon 3, 01.11.2023

FORESPØRSEL OM DELTAGELSE I FORSKNINGSPROSJEKTET

«Effekt av et selvmestringsprogram (self-management) sammenlignet med vanlig behandling for smerter på utsiden av hoften»

FORMÅLET MED PROSJEKTET OG HVORFOR DU BLIR SPURT

Dette er et spørsmål til deg om å delta i et forskningsprosjekt som undersøker effekten av selvmestring (self-management) for smerter på utsiden av hoften (laterale hoftesmerter). Deltakelse i prosjektet innebærer at du enten får opplæring i mestringsstrategier eller får vanlig behandling for smerter på utsiden av hoften. Vi ønsker i tillegg å undersøke dine erfaringer knyttet til det å leve med langvarige hoftesmerter både i hverdagslivet og i arbeidslivet. Prosjektet vil kunne bidra til ny kunnskap om behandling for personer med hoftesmerter.

Prosjektet vil bli utført ved Avdeling for fysikalsk medisin og rehabilitering, Oslo universitetssykehus (OUS), Ullevål.

HVA INNEBÆRER PROSJEKTET FOR DEG?

Smerter på utsiden av hoften er vanlig. Flere behandlingsmetoder har vært utprøvd, men man er fortsatt usikker på hvilken behandlingsmetode som har best virkning. Øvelsesbehandling har vist god effekt, både på kort og lang sikt, men det er usikkerhet rundt hvilke type øvelser, dosering og i hvilken form øvelsene best kan gis (veiledet av fysioterapeut eller egentrening). Tidligere studier har kun undersøkt veiledet trening. Informasjon og råd blir ofte gitt i kombinasjon med øvelser. Opplæring i selvmestring har ikke blitt undersøkt for hoftesmerter, men har vist god effekt på andre muskelskjelettplager. Annen behandling som brukes kan være trykkbølgebehandling, kortisoninjeksjoner, tøyninger og massasje. Ved vår avdeling består «vanlige behandling» av individuelt tilpasset informasjon og generelle smertelindrende råd, samt instruksjon i øvelser. Ofte anbefaler vi også oppfølging hos fastlege, fysioterapeut eller andre behandlere. Vi ønsker i dette prosjektet å undersøke om opplæring i et selvmestringsprogram gir bedre effekt på smerter og funksjon enn vanlig behandling.

Deltagere i prosjektet vil bli tilfeldig fordelt til én av to grupper:

1) Opplæring i selvmestring (self-management)
2) Vanlig behandling

Før behandlingen starter vil vi registrere opplysninger om deg ved hjelp av spørreskjema som omhandler smerte, funksjon og generell helsetilstand, i tillegg til å samle inn bakgrunnsinformasjon bl.a. om alder, utdanning og arbeidsstatus. Vi vil også gjøre en klinisk undersøkelse av hoften.

Dersom du blir trukket til **1) selvmestringsprogram**, så får du en undersøkelse hos lege og fysioterapeut på OUS med opplæring i hvordan du kan mestre smerte og symptomer og selv regulere aktivitet og øvelser. Du vil ha mulighet for 3-5 behandlingstimer i løpet av 12 ukers oppfølging ved OUS. Dersom du blir trukket til **2) vanlig behandling**, vil du få en undersøkelse hos lege og fysioterapeut på OUS, og ha mulighet til behandling i primærhelsetjenesten. Antall behandlinger og type tiltak bestemmes av den enkelte studiedeltager.

Alle deltagere vil få muntlig og skriftlig informasjon om smerter på utsiden av hoften, uavhengig av hvilken gruppe du blir trukket til.

Alle deltagere vil bli innkalt til kontrolltime hos lege ved OUS etter avsluttet behandling (6 måneder etter oppstart i studien). Da vil du også bli bedt om å svare på et elektronisk spørreskjema. Etter 12 måneder vil du få tilsendt et elektronisk spørreskjema på e-post/via SMS.

Samtykke til å delta i studien innebærer også deltakelse i en intervjusamtale med en fysioterapeut på OUS. Målet med intervju-studien er å få mer inngående kunnskap om hvordan du opplever å leve med hoftesmerter. Intervjuet vil ta mellom 30-60 minutter og vil foregå i etterkant av behandlingen du skal få.

MULIGE FORDELER OG ULEMPER

Økt kunnskap om smerter på utsiden av hoften, og strategier for å mestre hverdagen med langvarige smerter, kan ha en positiv innvirkning på aktivitet og deltagelse i dagliglivet.

Det er ikke kjent at treningsprogram ved hoftesmerter er forbundet med noen risiko for skade. Øvelser kan gi muskelstølhet og/eller smerter i og rundt hoften, men disse er som oftest forbigående. Det samme vil kunne gjelde dersom du prøver ut eller gjenopptar jobb- eller fritidsaktiviteter. Du vil bli fulgt opp underveis av fysioterapeut som du kan melde fra til ved eventuell forverring av plagene. Intervjusamtalen kan bidra til at du får mer innsikt i hva hoftesmertene og behandlingen innebærer for deg.

Behandlingen man får i dette prosjektet er behandling som man vanligvis vil kunne få hos en fysioterapeut, og som følger vanlige retningslinjer, og utgjør dermed ingen ekstra ulemper for den enkelte deltager.

FRIVILLIG DELTAKELSE OG MULIGHET FOR Å TREKKE DITT SAMTYKKE

Det er frivillig å delta i prosjektet. Dersom du ønsker å delta, undertegner du samtykkeerklæringen på siste side. Du kan når som helst og uten å oppgi noen grunn trekke ditt samtykke. Det vil ikke ha noen negative konsekvenser for deg eller din behandling hvis du ikke vil delta eller senere velger å trekke deg. Adgangen til å kreve sletting eller utlevering gjelder ikke dersom opplysningene er anonymisert eller publisert. Denne adgangen kan også begrenses dersom opplysningene har inngått i utførte analyser.

Dersom du senere ønsker å trekke deg eller har spørsmål til prosjektet, kan du kontakte prosjektleder (se kontaktinformasjon på siste side).

HVA SKJER MED OPPLYSNINGENE OM DEG?

Opplysningene som registreres om deg skal kun brukes slik som beskrevet under formålet med prosjektet, og planlegges brukt til 2035. Eventuell utvidelse i bruk og oppbevaringstid kan kun skje etter godkjenning fra REK (Regionale komiteer for medisinsk og helsefaglig forskningsetikk) og andre relevante myndigheter. Du har rett til innsyn i hvilke opplysninger som er registrert om deg og rett til å få korrigert eventuelle feil i de opplysningene som er registrert. Du har også rett til å få innsyn i sikkerhetstiltakene ved behandling av opplysningene. Du kan klage på behandlingen av dine opplysninger til Datatilsynet og institusjonen (Oslo universitetssykehus) sitt personvernombud.

Alle opplysningene vil bli behandlet uten navn og fødselsnummer eller andre direkte gjenkjennende opplysninger (=kodede opplysninger). En kode knytter deg til dine opplysninger gjennom en navneliste. Det er kun prosjektleder, Marianne Bakke Johnsen, stipendiat, Thea Morin Melås, og lege, Maren Lunder Wefring, som har tilgang til denne listen.

All publisering gjøres slik at enkeltdeltakere ikke kan gjenkjennes. Etter at forskningsprosjektet er ferdig, vil opplysningene om deg bli oppbevart i fem år av kontrollhensyn.

FORSIKRING

I studier med intervensjon som kan innebære risiko for skade så er det NPE (Norsk Pasientskadeerstatning) som gjelder dersom det er helsepersonell involvert i implementeringen av behandlingen, eller hvis implementeringen skjer på en helseinstitusjon.

ØKONOMI

Alle behandlingsalternativene benyttes i klinisk praksis og du betaler egenandel for behandlingen både hos fysioterapeut og lege ved OUS og i primærhelsetjenesten, med unntak av studiespesifikke kontroller (f.eks. ved inklusjon og kontroll etter endt behandling).

GODKJENNINGER

Regional komité for medisinsk og helsefaglig forskningsetikk (REK) har gjort en forskningsetisk vurdering og godkjent prosjektet (REK sør-øst 590816).

Oslo universitetssykehus og prosjektleder, Marianne Bakke Johnsen, er ansvarlig for personvernet i prosjektet.

Vi behandler opplysningene basert på ditt samtykke. Studien har rettslig grunnlag i generell personvernforordning artikkel 6 nr. 1 bokstav e) og artikkel 9 nr. 2 bokstav j). Det vil i tillegg innhentes samtykke.

KONTAKTOPPLYSNINGER

Dersom du har spørsmål til prosjektet, opplever uønskede hendelser eller bivirkninger, eller ønsker å trekke deg fra deltakelse, kan du kontakte prosjektleder, Marianne Bakke Johnsen, 22 11 97 14 / 67 23 86 49, m.b.johnsen@medisin.uio.no, eller stipendiat, Thea Morin Melås, 22 11 97 14.

Dersom du har spørsmål om personvernet i prosjektet, kan du kontakte personvernombudet ved Oslo universitetssykehus: personvern@oslo-universitetssykehus.no

JEG SAMTYKKER TIL Å DELTA I PROSJEKTET OG TIL AT MINE PERSONOPPLYSNINGER BRUKES SLIK DET ER BESKREVET

☐ Samtykke til forskningsdeltagelse.

☐ Samtykke til behandling av personopplysninger, herunder analyseresultater i gjennomføringen av studien.

Sted og dato

Deltakers signatur

Deltakers navn med trykte bokstaver
