# Supplementary material for: Self-management versus usual care for greater trochanteric pain syndrome (the HIPS trial): study protocol for a randomised controlled trial
Source: BMJ Open. 2025 Apr 5;15(4):e090688. doi: 10.1136/bmjopen-2024-090688 (PMC11973791; doi:10.1136/bmjopen-2024-090688)
Supplement: Supplementary file 3 [file bmjopen-2024-090688.reviewer_comments.pdf]

PEER REVIEW HISTORY

BMJ Open publishes all reviews undertaken for accepted manuscripts. Reviewers are asked to complete a checklist review form and are provided with free text boxes to elaborate on their assessment. These free text comments are reproduced below.

ARTICLE DETAILS

Title (Provisional)

Self-management versus usual care for greater trochanteric pain syndrome (the HIPS trial): study protocol for a randomized controlled trial

Authors

Morin Melås, Thea; Bjørneboe, John; Juel, Niels Gunnar; Wefring, Maren Lunder; Skatteboe, Sigrid; Killingmo, Rikke Munk; Engebretsen, Kaia B; Rathleff, Michael Skovdal; Øiestad, Britt Elin; Søberg, Helene Lundgaard; Pripp, Are Hugo; Brox, Jens Ivar; Johnsen, Marianne Bakke

---

VERSION 1 - REVIEW

**Reviewer**	**1**
**Name**	**Puntumetakul, Rungthip**
**Affiliation**	**Khon Kaen University, School fo Physical Therapy**
**Date**	**01-Sep-2024**
**COI**	**I declare that I have no known competing interests.**

Abstract

-Suggest providing conclusion in the abstract section.

Introduction

- It is still lacking the information why the authors think self-management would be superior to usual care in terms of pain and disability? Please spelling out a bit.
- Primary outcomes are Pain and Disability. I do not know why the authors chosen there two outcomes as the primary outcomes.
- Secondary outcomes are Fidelity, explore potential predictors, conduct mediation, and cost-effectiveness analysis. I do not know why the authors chosen there four outcomes as the secondary outcomes.

Methodology

- The authors recruited the participants, aged 18-70, that is very young. According to my knowledge the greater trochanteric pain syndrome often found in the older than younger groups, isn't it? Please explain more.
- Primary outcomes are Pain and Disability. I do not know why the authors chosen there two outcomes as the primary outcomes. Please explain more.
- For assessing the pain and disability, the authors stated that the participants rate

by themselves by using the VISA-G-Norwegian score at baseline, 3 and 6 months. Please confirm it this correct. If you say so, I think this would be one of the confounding variables.

- One of the inclusion criteria is pain intensity > 3 used the NRS, but the authors used the "VISA-G-Norwegian score" to assess pain and disability for assessing. It sounds not quite logical to me.

- I feel a bit awkward to put the inclusion and exclusion criterion in the Table. I think it occupied too much space.
- Why the authors used these inclusion and exclusion criteria. Please provide more details.

Results

-Wish to see a Table to demonstrate the demographic characteristic data of the subjects
in both groups.

- The authors said "Differences between groups in Quality Adjusted Life-Year (QALY) will be described with means (95% CI) and evaluated with ANCOVA", however, I do not see the Table demonstrating the results at all. I suggest the authors to demonstrate the results between the two groups comparing baseline to 3, 6 and 12 months.

- The Table 3, for me, is too difficult to follow. Please consider to rearrange.

Discussion

- I do not see the authors writing the discussion section. Why?

VERSION 1 - AUTHOR RESPONSE

Reviewers' comments:
Reviewer's name: Dr. Rungthip Puntumetakul

Comments to the Author:
Abstract
-Suggest providing conclusion in the abstract section.

Author response: *As the current manuscript is a study protocol for an ongoing trial, a conclusion cannot be reached at the current moment and is therefore not included in the abstract.*

Author action: *Edited the sentence regarding aims on page 2 in the introduction section of the abstract: "The aim of this study protocol is to describe a randomized controlled trial investigating the effectiveness of a self-management program versus usual care for patients with GTPS."*

And on page 3 line 29: "The main objective of this study protocol is to describe a randomized controlled trial comparing the effectiveness of a self-management program versus usual care for patients with GTPS."

Introduction

- It is still lacking the information why the authors think self-management would be superior to usual care in terms of pain and disability? Please spelling out a bit.

Author response:

Thank you for your comment. We know from previous studies that an active approach is recommended (1). However, there is currently no agreement on a superior treatment, and sham exercises have proven to be equally effective to specific exercises (2). This question if general activity is sufficient, and if education is an even more important component. The self-management intervention is a program with a cognitive behavioral context and targets improved function, reduced avoidance behaviors, and to build confidence to re-engage in meaningful activities. By identifying and utilizing resources through the five core skills (problem-solving, decision-making, resource utilization, therapeutic alliance, and taking action), self-management can support the patient in managing ongoing challenges, making it easier to adhere to an active approach as recommended. Therefore, we hypothesize that a self-management intervention, aimed at equipping patients with the confidence and skills to cope with challenges, may prove superior to usual care in terms of pain and disability. In addition, we argue that the persistent nature and impact of GTPS calls for a treatment that includes both physical- and psychosocial aspects and incorporates self-management strategies.

Author action: *Added* "..., which may prove beneficial in improving both pain and disability." to line 26 on page 3. The last part of the original sentence on line 25 "...but has yet to be investigated for patients with GTPS" is moved down to line 28 on the same page.

- Primary outcomes are Pain and Disability. I do not know why the authors chosen there two outcomes as the primary outcomes.

Author response: *We would like to clarify that there is only one primary outcome measure (VISA-G-Norwegian) that contains both pain and disability measures. The choice of primary outcome is related to the previously explained connection between self-management and pain and disability.*

- Secondary outcomes are Fidelity, explore potential predictors, conduct mediation, and cost-effectiveness analysis. I do not know why the authors chosen there four outcomes as the secondary outcomes.

Author response: *Evaluation of fidelity, exploration of potential predictors, mediation analysis, and cost-effectiveness are secondary aims of the study. Secondary outcomes are listed in Table 3 and include amongst others pain distribution, pain-related self-efficacy, and health-related quality of life.*

Methodology

- The authors recruited the participants, aged 18-70, that is very young. According to my knowledge the greater trochanteric pain syndrome often found in the older than younger groups, isn't it? Please explain more.

Author response: *We agree that GTPS is most common between 40-60 years of age, as mentioned in the introduction section. Even though the average patient most likely will be within this age group, we have both younger and older patients seeking care in our clinic. We argue that it would be wrong to exclude these as they also represent the patient population.*

- Primary outcomes are Pain and Disability. I do not know why the authors chosen there two outcomes as the primary outcomes. Please explain more.

Author response: *In our experience, challenges related to pain and disability are reasons why most GTPS patients seek help in the healthcare services, and therefore a measure that will most likely be considered relevant to improve. In addition, pain and disability are common outcomes in other studies of the current patient population, giving the opportunity to compare results.*

- For assessing the pain and disability, the authors stated that the participants rate by themselves by using the VISA-G-Norwegian score at baseline, 3 and 6 months. Please confirm it this correct. If you say so, I think this would be one of the confounding variables.

Author response: *We confirm that VISA-G-Norwegian will be the main outcome, which the participants fill out at baseline, 3, 6, and 12 months. This is a self-reported questionnaire, concerning pain and disability, which is validated for the current patient population. However, we agree that self-reported outcomes have weaknesses compared to more objective measures. Although, self-reported outcomes are recommended to enhance the patient perspective. When results from the study are published, these concerns will be addressed.*

Author action: *We have added "Only self-reported outcome measures" as a bullet point in the "strengths and limitations" section following the abstract on page 2.*

- One of the inclusion criteria is pain intensity > 3 used the NRS, but the authors used the "VISA-G-Norwegian score" to assess pain and disability for assessing. It sounds not quite logical to me.

Author response: *We understand that it might not be considered logical to include different measures of pain for inclusion and outcome. The intention behind the use of NRS at inclusion (reported as average the last week) is to ensure that the recruited patients currently experience pain to avoid floor effects. We acknowledge that the primary outcome VISA-G-Norwegian also includes measures of pain and disability, which could have been used as a criterion for inclusion. However, we chose a simpler pain measure to save time in the recruitment phase.*

- I feel a bit awkward to put the inclusion and exclusion criterion in the Table. I think it occupied too much space.

Author response: *We understand that the table takes up space, but we believe it enhances clarity and readability. Inclusion- and exclusion criteria are often depicted this way in a protocol for a randomized controlled trial, therefore we prefer to retain this format.*

- Why the authors used these inclusion and exclusion criteria. Please provide more details.

Author response: *The inclusion criteria ensure that we recruit the targeted population as intended. This includes verifying the clinical diagnosis, with reproduction of pain on palpation and at least one positive clinical provocation test. The different tests and palpation were chosen*

aligning other studies of the patient population (3). Exclusion criteria ensure that we are excluding differential diagnoses. Due to limited funding to interpreters in the project, the criterion "understanding oral and written Norwegian" make sure that participants can answer the self-reported questionnaires and follow the self-management intervention. The section entitled "baseline assessment" gives more details regarding this.

Author action: *Added on page 4 line 40* "A cut-off in pain intensity of at least 3 on a numeric rating scale (NRS) was set to ensure recruitment of patients that are currently experiencing symptoms."

Results

-Wish to see a Table to demonstrate the demographic characteristic data of the subjects in both groups.

Author response: *As this is a study protocol for an ongoing trial, we do not yet have any demographic characteristics to display. This will of course be a part of the article with the results of the study.*

- The authors said "Differences between groups in Quality Adjusted Life-Year (QALY) will be described with means (95% CI) and evaluated with ANCOVA", however, I do not see the Table demonstrating the results at all. I suggest the authors to demonstrate the results between the two groups comparing baseline to 3, 6 and 12 months.

Author response: *As mentioned earlier we are not able to display any results yet as this is an ongoing trial.*

- The Table 3, for me, is too difficult to follow. Please consider to rearrange.

Author response: *We appreciate that Table 3 is quite extensive and contains a lot of information. However, we think this table provides a valuable timeline concerning the different outcome measures and their purpose. We recognize that it could benefit from some simplification, and have therefore removed the interventions and baseline characteristics from the table. A description of baseline characteristics is inserted in the text above the table.*

Author action: *Added on page 10 line 199:* "*such as age, sex, education, work status, comorbidities, physical activity level, sleep disturbance, and menopausal status are included at baseline to describe the patient population.*"

Discussion

- I do not see the authors writing the discussion section. Why?

Author response**:** *We have adhered to the author guidelines provided by BMJ Open for protocol manuscripts with the recommended headlines. A discussion section will be provided in the article with results from the RCT.*

1. Kjeldsen T, Hvidt KJ, Bohn MB, Mygind-Klavsen B, Lind M, Semciw AI, et al. Exercise compared to a control condition or other conservative treatment options in patients with Greater Trochanteric Pain Syndrome: a systematic review and meta-analysis of randomized controlled trials. Physiotherapy. 2024;123:69-80.
2. Ganderton C, Semciw A, Cook J, Moreira E, Pizzari T. Gluteal Loading Versus Sham Exercises to Improve Pain and Dysfunction in Postmenopausal Women with Greater Trochanteric Pain Syndrome: A Randomized Controlled Trial. J Womens Health (Larchmt). 2018;27(6):815-29.
3. Mellor R, Bennell K, Grimaldi A, Nicolson P, Kasza J, Hodges P, et al. Education plus exercise versus corticosteroid injection use versus a wait and see approach on global outcome and pain from gluteal tendinopathy: prospective, single blinded, randomised clinical trial. Br J Sports Med. 2018;52(22):1464-72.
